# Metabolic activation and toxicological evaluation of polychlorinated biphenyls in *Drosophila melanogaster*

**DOI:** 10.1038/s41598-020-78405-z

**Published:** 2020-12-09

**Authors:** T. Idda, C. Bonas, J. Hoffmann, J. Bertram, N. Quinete, T. Schettgen, K. Fietkau, A. Esser, M. B. Stope, M. M. Leijs, J. M. Baron, T. Kraus, A. Voigt, P. Ziegler

**Affiliations:** 1 Institute for Occupational, Social and Environmental Medicine, RWTH Aachen University, Aachen, Germany; 2 Department of Chemistry and Biochemistry, Florida International University Florida, Florida, USA; 3 Department of Dermatology and Allergology, RWTH Aachen University, 52074 Aachen, Germany; 4 Department of Gynecology and Gynecological Oncology, University Hospital Bonn, Bonn, Germany; 5 Department of Neurology, University Medical Center, RWTH Aachen University, 52074 Aachen, Germany; 6 JARA-BRAIN Institute Molecular Neuroscience and Neuroimaging, Forschungszentrum Jülich GmbH, RWTH Aachen University, 52074 Aachen, Germany

**Keywords:** Cell biology, Health occupations

## Abstract

Degradation of polychlorinated biphenyls (PCBs) is initiated by cytochrome P450 (CYP) enzymes and includes PCB oxidation to OH-metabolites, which often display a higher toxicity than their parental compounds. In search of an animal model reflecting PCB metabolism and toxicity, we tested *Drosophila melanogaster*, a well-known model system for genetics and human disease. Feeding *Drosophila* with lower chlorinated (LC) PCB congeners 28, 52 or 101 resulted in the detection of a human-like pattern of respective OH-metabolites in fly lysates. Feeding flies high PCB 28 concentrations caused lethality. Thus we silenced selected CYPs via RNA interference and analyzed the effect on PCB 28-derived metabolite formation by assaying 3-OH-2′,4,4′-trichlorobiphenyl (3-OHCB 28) and 3′-OH-4′,4,6′-trichlorobiphenyl (3′-OHCB 28) in fly lysates. We identified several drosophila CYPs (dCYPs) whose knockdown reduced PCB 28-derived OH-metabolites and suppressed PCB 28 induced lethality including dCYP1A2. Following in vitro analysis using a liver-like CYP-cocktail, containing human orthologues of dCYP1A2, we confirm human CYP1A2 as a PCB 28 metabolizing enzyme. PCB 28-induced mortality in flies was accompanied by locomotor impairment, a common phenotype of neurodegenerative disorders. Along this line, we show PCB 28-initiated caspase activation in differentiated fly neurons. This suggested the loss of neurons through apoptosis. Our findings in flies are congruent with observation in human exposed to high PCB levels. In plasma samples of PCB exposed humans, levels of the neurofilament light chain increase after LC-PCB exposure, indicating neuronal damage. In summary our findings demonstrate parallels between *Drosophila* and the human systems with respect to CYP mediated metabolism and PCB mediated neurotoxicity.

## Introduction

The International Agency for Research on Cancer (IARC) has classified polychlorinated biphenyls (PCBs) as carcinogenic to humans (Group 1)^1^. IARC also points to the importance of metabolic activation for the toxic and carcinogenic effects of PCBs^2^. Our own work shows genotoxic and thus potentially carcinogenic effects of activated PCB 28 metabolites in vitro^3^. The metabolic activation of PCBs is initiated by cytochrome p450 (CYP) family members and includes the oxidation of PCBs to OH-PCBs. OH-PCBs are expected to be readily conjugated and excreted, however some OH-PCBs have been shown to be strongly retained in human blood by enterohepatic circulation^4,5^. OH-PCBs can potentially reach any hydrophilic compartment in the body, where they are oxidized in peripheral tissues by enzymatic catalysis (e.g. myeloperoxidase, prostaglandin-H-synthase) or autoxidized to catechols or hydroquinones, which after further oxidation to quinones and semichinones can covalently bind to biological macromolecules such as purine bases or proteins (adduct formation)^6–8^. Compared to their parental compounds OH-PCBs often show equal or higher toxicity, greater distribution in the human body and longer half-lives^9,10^. With the CYP- dependent metabolism of PCBs, excessive amounts of reactive oxygene species (ROS) can be generated, which mediate cytotoxic effects. It has been shown that PCBs cause oxidative stress in nerve cell cultures as well as in the hippocampus, the cerebral cortex and the cerebellum of rats^11,12^, which in turn induces apoptosis and necrosis. In PCB treated rats, ROS oxidized proteins to protein-carbonylene and inhibited the function of the enzyme acetylcholine esterase and a number of other enzymes involved in the elimination of oxygen radicals^13^. In the same study, an accumulation of hydrogen peroxide and an increased lipid peroxidation were demonstrated. These results suggested that reactive intermediates of the PCB metabolism trigger neurodegenerative processes in the brain, which can eventually lead to the loss of neurons. These findings are supported by the fact, that behavioral studies in animals show that PCBs influence learning behavior, memory and fine motor skills^14^. Similar effects are documented in humans after accidental exposure to PCBs^15–18^.

To predict adverse effects of PCB exposure on humans, simple animal models that reflect the human situation as accurately as possible are invaluable. We therefore asked whether *Drosophila melanogaster* might be a suitable model to analyze the metabolism of lower chlorinated PCBs and the associated toxicity. We knocked down selected Cyps in *Drosophila* (dCyps) using RNA interference (RNAi) in order to identify isoenzymes responsible for the conversion of the indicator congener 2,4,4′-trichlorobiphenyl (PCB 28) in vivo. To test whether human orthologs of identified dCyps were involved in PCB28 hydroxylation we used a human liver-like CYP-cocktail. Finally, we investigated the PCB 28-mediated lethality in *Drosophila* using the neuron-specific expression of an apoptosis-sensitive biosensor.

## Material and methods

### Fly stocks

Flies were raised and maintained on standard cornmeal-yeast medium under a 12/12 h light–dark cycle at 25 °C. The fly stocks used were obtained either from the Vienna *Drosophila* Resource Center (VDRC, www.vdrc.at), or the Bloomington *Drosophila* Stock Center (BDSC, www.bdsc.indiana.edu). Strains with GAL4-dependent expression of short hairpins (sh) to induce gene silencing via RNA interference (RNAi) of the following genes were used: sh-Cyp4ac1 (BDSC #67005), sh-Cyp307a2 (VDRC #330647), sh-Cyp18a1 (BDSC #64923), sh-Cyp9f2 (BDSC #67806), sh-Cyp4d2 (BDSC #42600), sh-Cyp4d1 (BDSC #52979), sh-Cyp311a1 (BDSC #67792), sh-Cyp312a1 (VDRC #101231), sh-Cyp28d2 (BDSC #61282), sh-Cyp28d1 (BDSC #53892), sh-Cyp4d21 (BDSC # 29238), sh-Cyp28a5 (BDSC #77405), sh-4d20 (BDSC #77341), sh-Cyp4s3 (BDSC #66978), sh-Cyp4c3 (BDSC #64213), h-Cyp304-a1 (BDSC #67805). Canton-S (BDSC #64349) served as wild type control. GAL4 drivers used to activate UAS-controlled expression of above mentioned sh-lines were actin-GAL4 (BDSC #4414) and daughterless-GAL4 (BDSC #55850) for ubiquitous expression. The driver elav-GAL4 (BDSC #458) was used to induce pan neural expression and/or GMR-GAL4 (BDSC #1104) to induce retina specific expression of transgenes under UAS-control. UAS-Apoliner (BDSC #32122) was used to detect caspase activation in flies after PCB 28 treatment.

### Fly crossing

In genomic profiling experiments, ubiquitous expression of shRNA to silence certain Cyps was achieved by crossing virgin act-GAL4 driver females with male flies of the respective sh-RNA strain. In the F1 generation, carriers of the ubiquitous Cyp knockdown were selected and used for experiments. To investigate the influence of certain Cyps on PCB 28-mediated neurotoxicity, we used Apoliner, a biosensor for caspase activation and thus an indicator of apoptosis. Neuronal specific expression of the caspase biosensor was achieved by pairing parental Apoliner line UAS-GMR strains with the neuronal driver strain elav-GAL4. Age matched females were transferred to PCB 28-containing food and analyzed for the induction of apoptosis by flow cytometry.

### PCB administration

PCBs were administered through artificial fly food (2% agar, 2% LB broth, 4% sucrose) to flies. For initial experiments food containing 100 µM PCB (PCB 28, PCB 101 or PCB 52) was feed to flies to determine lethality. For metabolism screening, 20 µM PCB were administered via the fly food. Lethality screenings with Cyp knockdowns were performed using a 60 µM PCB 28 concentration in the fly food and viability was assayed after 48 h. Metabolism screenings were performed with a sublethal concentration of 20 µM PCB 28 for 24 h. Lethality screenings with Apoliner caspase biosensor flies were performed on 10 to 40 µM PCB 28 for 24 h. For detection of free GFP by flow cytometry, the duration of the PCB treatment was reduced to 4 h.

### Analysis of PCB metabolites by online solid phase extraction (SPE) method coupled to liquid chromatography-tandem mass spectrometry (LC–MS/MS)

Male flies (≤ 5 days post eclusion) were transferred to artificial fly food containing sub-lethal concentrations of PCBs as indicated. After 24 h on PCB food, flies were killed by freezing (freezer − 25 °C; 15 min) and washed with PBS to remove residual PCB. Fly lysates were generated in RIPA buffer by mechanical disruption in a speed mill (Analytic Jena) using ceramic beads. Lysates were cleared of exoskeleton and cell debris by centrifugation. For analytical preparations, *Drosophila* lysates were prepared following the procedures previously reported in^19^. In brief, *Drosophila* lysate (equivalent of 12 flies) were diluted 1:2 with 80 µL of ammonium acetate buffer 0.1 mol L^−1^ (pH = 5.3). 100 µL of this dilution were further incubated with 100 µL of ammonium acetate buffer 0.1 mol L^−1^ (pH = 5.3) and 5 µL of ß-Glucuronidase/Arylsulfatase enzyme overnight in a drying oven at 37 °C for enzymatic hydrolysis in order to release conjugated compounds. 50 µL of a mix of internal standards (10 ng mL^−1^) and 600 µL of methanol were added to the samples, then mixed by vortexing for 1 min and centrifuged for 10 min at 4500 rpm for protein precipitation. The individual supernatants were transferred to glass LC vials and evaporated until approximately 50 µL at 45 °C under a gentle stream of nitrogen. Finally, 0.1 mol L^−1^ ammonium acetate buffer was added to a final volume of 100 µL and then transferred to an insert for analysis. The online solid phase extraction (SPE) method coupled to liquid chromatography-tandem mass spectrometry (LC–MS/MS) has been previously described by Quinete^19^ and was carried out using an API 5500 QTrapmass spectrometer (AB Sciex, Darmstadt, Germany) equipped with electrospray ionization (ESI) interface.

### Incubation of PCB 28 with a liver-like CYP-cocktail

Recombinantly expressed CYP-bactosomes (coexpressed with CYP-reductase in Escherichia coli) were obtained from Tebu-Bio (Offenbach, Germany) and mixed in the concentrations described^20,21^. Incubations contained PCB 28 (100 µM), HEPES buffer (50 mM pH 7.4), MgCl_2_ (30 mM) and NADPH (1 mM) in a total volume of 500 µL. Incubations were performed in triplicates and initialized by adding NADPH after 3 min of pre-incubation at 37 °C and terminated after 60 min. Control samples were run in the absence of substrate, in the absence of NADPH or using heat-deactivated enzymes. Analysis was performed using GC/MS and LC/MS as described^22^.

### Detection of cleaved GFP-fragment by Western-blot and flow cytometry

Male flies with pan neural and retinal expression of the caspase biosensor Apoliner were kept on artificial fly food containing increasing concentrations of PCB 28 as indicated. Males elav-GAL4 flies without expression of the caspase sensor served as controls. After 4 h of incubation, vital flies were anaesthetised with CO_2_ and the heads prepared. Head lysates were generated in PBS with 2% FCS by mechanical disruption in a shaker with the support of glass beads. Lysates were passed through a cell sieve (70 μm) and centrifuged up to 12,000×*g* for a few seconds. The supernatant was discarded and the cell pellet was resolved in 250 μL PBS with 2% FCS for analysis by flow cytometry at FACSCanto II (BD, Franklin Lakes NJ, USA). For Western-blotting, head lysates were generated in RIPA buffer (10 µl per fly head) by mechanical disruption in a bead ruptor with the support of ceramic beads and subsequent centrifugation of exoskeleton and cell debris. For immune-blotting, proteins were separated by SDS-PAGE and transferred to a 0.2 µm Trans-Blot Turbo Mini PVDF Membran (BioRad, Hercules, CA, USA) according to the manufacturer's specifications. The membrane was blocked with a TBST buffer (200 mM Tris(hydroxymethyl)aminomethane, 1.5 M NaCl, 1% (v/v) Tween-20, pH 7.5) containing 5% milk powder for 1 h at room temperature and incubated with the primary anti-GFP antibody (Cat. # ab6556, Abcam, Cambridge, UK) diluted 1:5,000 overnight at 4 °C. The membrane was washed three times with a TBST buffer for 15 min and treated with a suitable secondary anti-rabbit IgG antibody conjugated to horseradish peroxidase (Cat. # ab205718, Abcam, Cambridge, UK) diluted 1:10,000 for 1 h at room temperature. β-Actin served as control and was detected with an primary anti-β-Actin antibody (JLA20, DSHB, Iowa, IA, USA) diluted 1:1000 and a suitable secondary anti-mouse IgG antibody conjugated to horseradish peroxidase (Cat. # ab205719, Abcam, Cambridge, UK) diluted 1:1000 for 1 h. Bound antibodies were detected by chemiluminescence using and ECL-kit according to manufacturers instructions (ECL, Merck Millipore, Burlington, MA, USA).

### Neurofilament 3 ELISA

Neurofilament 3 content in plasma samples of the HELPcB-Cohort (Kraus et al. 2012) was determined using a commercial ELISA Kit (Human Neurofilament 3 ELISA Kit Elabscience, Houston, TX, USA) according to the manufacturer's specifications. Plasma was diluted 1:5 with sample dilution buffer and subsequently 100 µL were used for the ELISA. Samples from the same donors were taken at two different time points (2011 and 2015).

### Statistical analysis

Results are given as mean values ±< standard deviation of at least three technical replicates or three biological replicates as indicated in the respective figure. In all fly experiments, one biological replica corresponds to 12 flies. All analysis were conducted with the software SAS 9.4^23^. We used the GLIMMIX procedure to conduct an ANOVA-test where indicated. The distribution of the dependent variables if metric were observed by histograms, Q-Q-plots and Shapiro–Wilk and Komolgorov-Smirnov tests. A post hoc Tukey-test was applied to account for multiple comparison. If the independent variable was metric, we tested the covariance between the groups and adjusted for inhomogeneous variances if required. Unless otherwise noted, significances in the figures are as *p ≤ 0.05; **p ≤ 0.01; ***p ≤ 0.005; ****p ≤ 0.0001. Non-significant differences are not indicated.

## Results

### Bioactivation of PCB 28, PCB 101 and PCB 52 in *Drosophila melanogaster*

**Figure 1:**
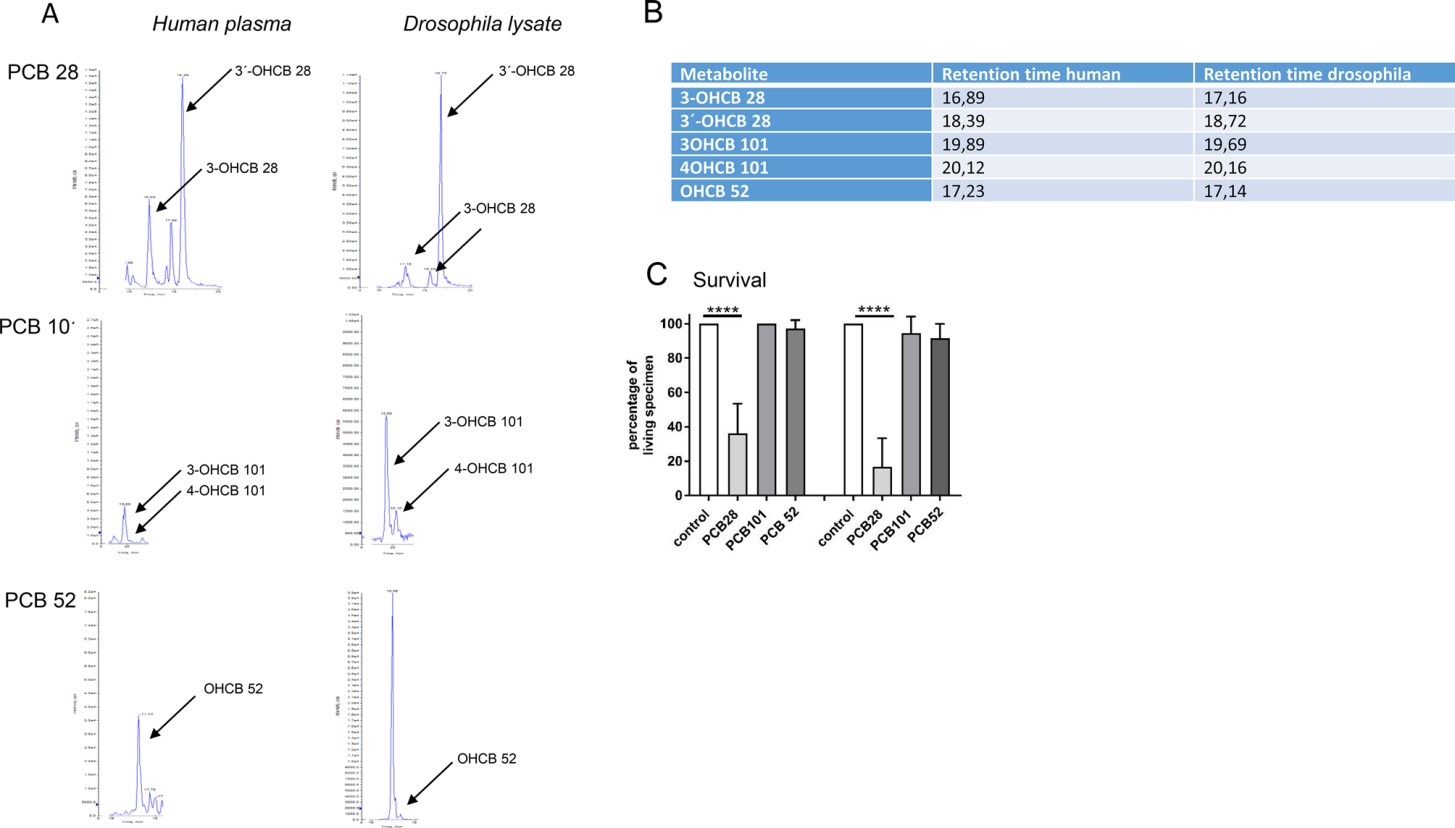
Cytochrome P450 monooxygenase-dependent metabolism of PCB 28, PCB 101 and PCB 52 in humans and in *Drosophila melanogaster*. (**A**) LC–MS/MS chromatogram of 3-OH-2′,4,4′-trichlorobiphenyl (3-OHCB 28), 3′-OH-4′,4,6′-trichlorobiphenyl (3´-OHCB 28), 3-OH-2,2′,4′,5,5′-pentachlorobiphenyl (3-OHCB 101), 4-OH-2,2′,4′,5,5′-pentachlorobiphenyl (4-OHCB 101) and 4-OH-2,2′,5,5′-tetrachlorobiphenyl (4-OHCB 52). Left panel, representative samples from individuals occupationally exposed to high levels of the respective PCB. Right panel, whole fly lysates from *Drosophila melanogaster* which were fed with a low dose (20 µM) of the respective PCBs for 24 h. One representative sample from several experiments is shown. (**B**) Corresponding retention times of targeted metabolites in human plasma and *Drosophila melanogaster* as shown in 1A. (**C**) Survival of *Drosophila melanogaster* fed with a high dose (100 µM) of PCB 28, PCB 101 and PCB 52 for 48 h and 72 h. Mean ± SD of 3 different biological experiments each with 3 technical replicates are shown (each technical replicate corresponds to 12 flies). Differences were calculated by repeated measurement ANOVA and posthoc Tukey-test. Statistical differences for PCB 28 versus control (48 h) and for 72 h were p ≤ 0.0001.

We have recently identified more than 20 OH-PCBs congeners in human plasma samples from individuals with high PCB body exposure due to occupational exposure in a transformer recycling company^24^. Congeners included metabolites of PCB 28: 3-OH-2′,4,4′-trichlorobiphenyl (3-OHCB 28), 3′-OH-4′,4,6′-trichlorobiphenyl (3′-OHCB 28); metabolites of PCB 101: 3-OH-2,2′,4′,5,5′-pentachlorobiphenyl (3-OHCB 101), 4-OH-2,2′,4′,5,5′-pentachlorobiphenyl (4-OHCB 101) and one metabolite of PCB 52: 4-OH-2,2′,5,5′-tetrachlorobiphenyl (4-OHCB 52) (Fig. 1A, left panel). In a proof of principle experiment, we determined PCB 28, PCB 101 and PCB 52 metabolization in *Drosophila melanogaster* (Fig. 1A, right panel). After flies were fed for 24 h with PCB congeners (agar concentration 20 µM), a human-like pattern of respective OH-metabolites was detected in *Drosophila* whole body lysates. Chromatographic retention times of OH-metabolites detected in *Drosophila* showed no difference to internal standards and corresponded to OH-metabolites in human plasma samples (3-OHCB 28: 17.16 vs. 16.89; 3′-OHCB 28: 18.72 vs. 18.39; 3-OHCB 101 19.69 vs. 19.89; 4-OHCB 101: 20.16 vs. 20.12 and 4-OHCB 52: 17.14 vs.17.23, Fig. 1B), suggesting the formation of identical metabolites in both organisms. By feeding different concentrations of parental PCBs to *Drosophila*, we could demonstrate high mortality at an agar concentration of 100 µM PCB 28. Interestingly, feeding PCB 101 and PCB 52 at an agar concentration of 100 µM did not result in lethality (Fig. 1C). As synthetic OH-metabolites of PCB 28-induced cytotoxicity in a model cell culture system^3^, we conclude that PCB 28 bioactivation by cytochrome p450 (Cyp) enzymes leads to toxicity in *Drosophila.* These results further suggest that the formation of OH-metabolites from parental PCBs has been partially preserved during evolution and that *Drosophila melanogaster* is an attractive model for the bioactivation of PCBs by Cyp enzymes, providing a useful basis for the development of more accurate bioassays.

### PCB 28 metabolism: a targeted genetic screen using flies with RNAi-mediated knockdown of cytochrome p450 enzymes

**Figure 2:**
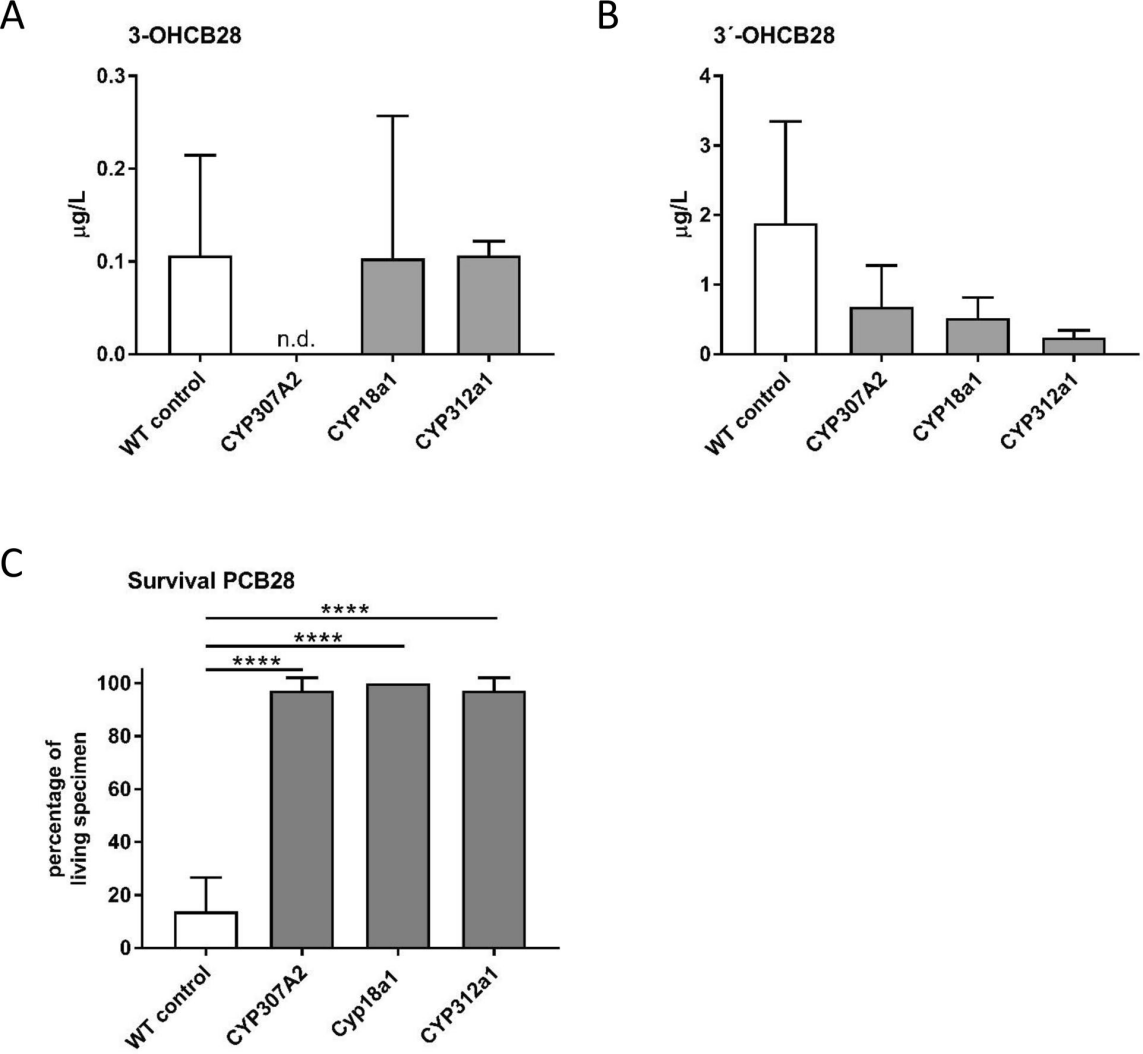
Metabolism of PCB 28 in *Drosophila* pairings with knockdowns in either CYP307a2, 18a1 or 312a1 compared to WT Canton-S. Flies were generated and treated as described in Table 1. The amount of 3-OH-CB28 (**A**) and 3´-OH-CB28 (**B**) in WT Canton-S or *Drosophila* with knockdowns of Cyp307a2, Cyp18a1 or Cyp312a1. (**C**) Survival rate of the respective *Drosophila* specimen at high PCB 28 concentrations. Mean ± SD of 3 different biological experiments each with 3 technical replicates are shown (each technical replicate corresponds to 12 flies). Differences were calculated by repeated measurement ANOVA and posthoc Tukey-test. (**A**,**B**) differences are not significant. Figure C control versus knockdowns of Cyp307a2, Cyp18a1 or Cyp312a1; p ≤ 0.0001.

In order to identify which dCYP enzymes are involved in the bioactivation of PCB 28, we expressed short hairpin (sh)RNA to induce RNAi-mediated gene silencing of individual CYP coding genes. In total, we knocked down the expression of 19 individual Cyp P450 genes and assayed the effect on PCB 28 metabolite formation (Table 1). The individual dCyp P450 genes were selected based on the presence of human orthologs, the availability of shRNA fly strains and the involvement of the human ortholog in the action of xenobiotics. Emphasis was placed on involving orthologs, which are collectively responsible for the catalysis of almost 75% of all known phase I drug oxidation reactions in humans. Male flies with ubiquitous (driver-) silencing of an individual Cyp P450 gene were fed PCB 28 at low (20 µM) concentrations for 24 h. Subsequently, fly lysates were analyzed for abundance of PCB 28 metabolites. In 18 knockdowns the uptake of the parental PCB28 was detected on a regular basis (data not shown). One knockdown (CYP303a1, human orthologue CYP2B6) never showed the uptake of PCB 28 and was therefore excluded from further analysis. In addition, the uptake of PCB28 varied widely (up to a logarithmic level) between knockdowns, and this level of variation was not reflected by the amount of metabolites which were produced. We therefore decided to analyse the relative amount of metabolites produced in each knockdown in comparison to a control group. In most of the knockdowns analyzed by LC–MS/MS the relative amount of the metabolite 3-OHCB28 was higher than in the control group (n-13), with only 5 knockdowns showing reduced levels. For the metabolite 3′-OHCB28 only 3 knockdowns showed a higher production than the control group, while 15 knockdowns produced a lower amount of this metabolite (Table 1). To identify knockdowns that were highly likely to be involved in the metabolism of PCB 28, we decided to look for knockdowns that did either not produce any of the main metabolites at all or produced less of both metabolites simultaneously than the control group. Our analysis with LC–MS/MS revealed that silencing of three Cyp genes (Cyp307a2, 18a1 and 312a1) caused a reduced abundance of the both metabolites 3-OHCB 28 and 3′-OHCB 28, respectively (Fig. 2A,B). In flies with RNAi-mediated silencing of Cyp307a2 (human orthologs: CYP1A1 and CYP1A2), the metabolite 3-OHCB 28 was not detected and 3′-OHCB 28 was reduced by more than 70% compared to control levels. When Cyp18a1 (human orthologues: CYP2C19, CYP2C8, CYP2C9, CYP2J2, CYP2R1, CYP21A2, CYP2A6, CYP2D6, CYP2 F1, CYP2S1, CYP2U1 and CYP2E1) was silenced, a 10% reduction of 3-OHCB 28 and a 70% reduction of 3´-OHCB 28 was observed compared to control. Similarely, silencing of Cyp312a1 (human orthologue: CYP51A1) resulted in a mild (10%) reduction of 3-OHCB 28 and a strong (90%) reduction of 3´-OHCB 28. At high PCB28 concentrations (agar concentration of 100 µM), RNAi-mediated silencing of Cyp307a2, 18a1 and 312a1 resulted in a low mortality rate (Fig. 2C), while only roughly 15% of the control flies survived. These results suggested, that the knockdown of Cyp307a2, 18a1 and 312a1 in *Drosophila* leads to a lower production of 3´-OHCB 28 and 3-OHCB 28,which might explain the better survival of flies with RNAi-mediated silencing of respective Cyp genes in the presence of PCB 28.

**Table 1:** PCB 28 metabolisation and mortality of adult *Drosophila* wild type (WT) and genotypes with an ubiquitous expression of dsRNA for certain CYP enzymes.

Human CYP	*Drosophila* orthologue	BDSC ID (* VDRC ID)	3-OHCB 28 (µg/L)	3´-OHCB 28 (µg/L)	3-OHCB 28% of control	3´-OHCB 28% of control
Control	WT Canton-S	64349	0.11	1.88	100	100
CYP19A1, CYP4F8, CYP4F11, CYP4F12	Cyp4ac1 (act-GAL4)	67005	0.23	3.69	209. 1	196.2
**CYP1A1/ CYP1A2**	Cyp307a2 (dag-GAL4)	330647*	Not detected	0.69	0	36.7
**CYP2C19**, **CYP2C8**, **CYP2C9**, CYP2J2, CYP2R1, CYP21A2, **CYP2A6**, CYP2A13, CYP2C18, **CYP2D6**, CYP2F1, CYP2S1, CYP2U1, **CYP2E1**	Cyp18a1 (act-GAL4)	64923	0.10	0.52	90.9	27.7
**CYP3A4**, CYP3A43	Cyp9f2 (act-GAL4)	67806	0.10	1.40	95.4	74.5
CYP4F2, CYP4F3	Cyp4d2 (act-GAL4)	42600	0.18	0.70	163.6	37.2
CYP4X1	Cyp4d1 (dag-GAL4)	52979	0.15	1.47	136.4	78.2
CYP4Z1, CYP4B1	Cyp311a1 (act-GAL4)	67792	0.23	1.56	209.1	82.9
CYP51A1	Cyp312a1 (act-GAL4)	101231*	0.10	0.24	90.9	12.7
CYP7A1	Cyp28d2 (act-GAL4)	61282	0.26	1.55	236.4	82.4
CYP7B1	Cyp28d1 (act-GAL4)	53892	0.09	3.97	81.1	211.2
CYP46A1	Cyp4ad1 (act-GAL4)	61745	0.26	1.26	236.4	67
CYP39A1	Cyp4d21 (act-GAL4)	29238	0.25	1.84	227.3	97.9
CYP8A1	Cyp28a5 (act-GAL4)	77405	0.29	1.57	263.4	83.5
CYP26C1	Cyp4d20 (act-GAL4)	77341	0.37	0.31	336.4	16.5
CYP4A22	Cyp4s3 (act-GAL4)	66978	0.17	1.68	154.5	89.4
CYP4V2	Cyp4c3 (act-GAL4)	64213	0.21	1.05	190.9	55.85
**CYP3A5**	Cyp9b1 (dag-GAL4)	68126	0.19	1.93	172.7	102.6
CYP17A1	Cyp304a1 (dag-GAL4)	67805	0.28	0.29	254.5	15.4
CYP2B6	Cyp303a1 (dag-GAL4)	51716	Not determined	Not determined	Not determined	Not determined

Male flies of the respective RNAi strain (UAS-CYP) were paired with virgins of the ubiquitous driver strain act-GAL4 or dag-GAL4as indicated. For metabolism screening, F1 generations were fed with PCB 28 at low (20 µM) concentrations. After 48 h of incubation the amount of 3-OH-CB28 and 3´-OH-CB28 in flies was determined using LC–MS/MS and a 9-point calibration curve as described^24^. Mean of 3 technical replicates each with a total of 12 flies per replicate are shown.

### Metabolic activation of PCB 28 by a human liver-like cytochrome P450 cocktail.

**Figure 3:**
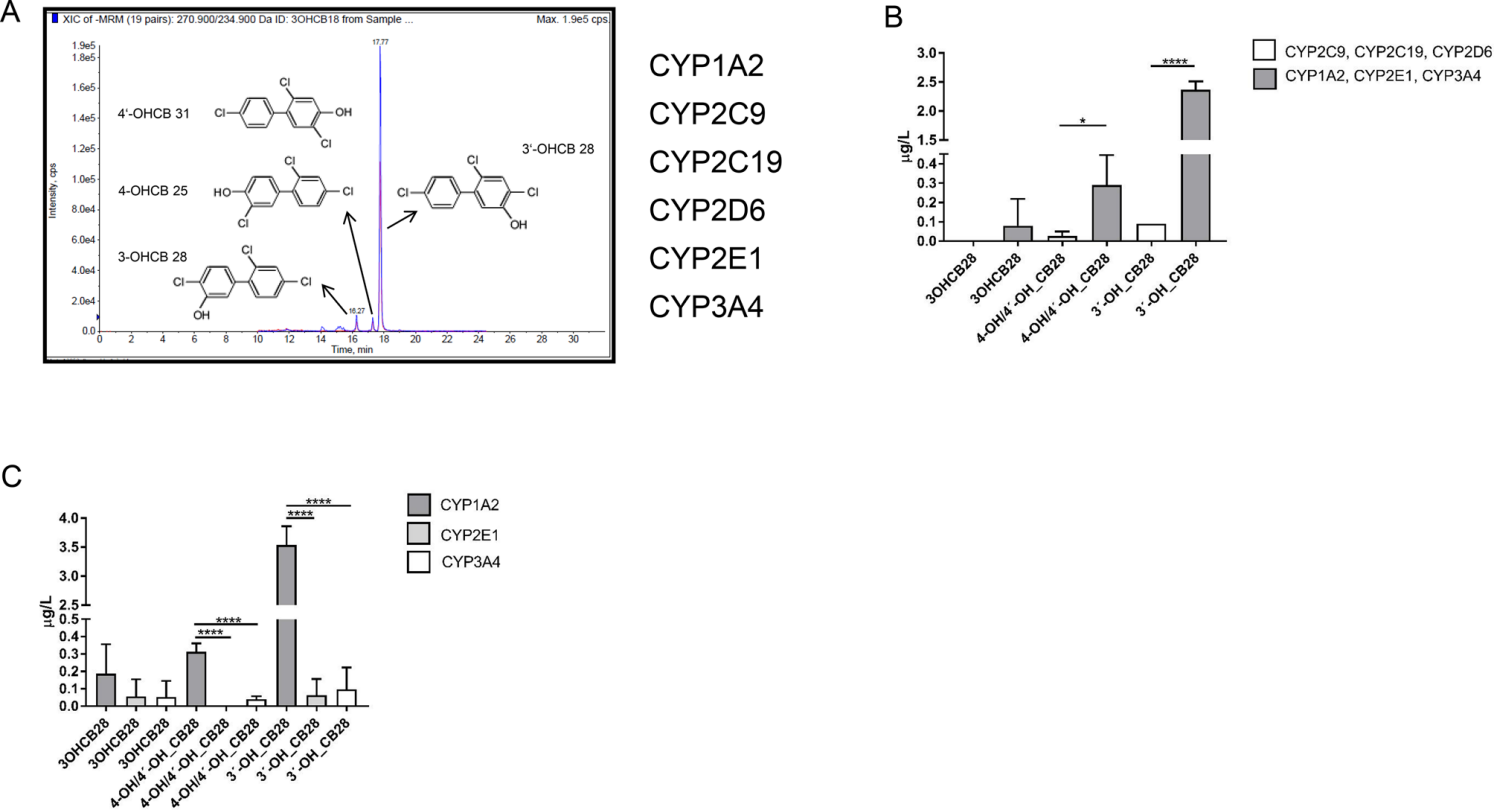
Metabolic activation of PCB 28 in the presence of a recombinant liver-like cytochrome P450 cocktail. (**A**) LC–MS/MS chromatogram of 3-OH-CB28, 3´-OH-CB28 and 4-OH/4´-OH-CB28 after incubation of PCB 28 with recombinantly expressed CYP1A2, CYP2C9, CYP2C19, CYP2D6, CYP2E1 and CYP3A4 (rhCYP cocktail) for 1 h. (**B**) Amounts of 3-OHCB 28, 3´-OHCB 28 and 4-OH/4´-OHCB 28 detected after incubation of PCB 28 with different constituents of the rhCYP cocktail. (**C**) Amounts of OH-CB28 metabolites detected after single incubation of PCB 28 either with CYP1A2, CYP2E1 or CYP3A4. Mean ± SD of 3 technical replicates are shown. (**B**,**C**) Results between groups of CYPs were compared by ANOVA and adjusted by posthoc Tukey-test for multiple measures. Statistical differences are as indicated (*p ≤ 0.05; ****p ≤ 0.0001).

The CYP enzymes responsible for the metabolism of PCB28 in humans have not yet been described. However, there are indications that these could be CYP enzymes that are expressed in the liver^25^. Taking these results into account, we decided to subject PCB 28 to in vitro metabolism by a human liver-like cytochrome P450 cocktail (rhCYP cocktail)^20,21^. This system allowed us to simultaneously test whether the human orthologs of the dCYP enzymes identified within our in vivo screening were also involved in metabolizing PCB 28 (Table 1). The liver-like rhCYP cocktail consists of the main liver enzyme CYP1A2 (drosophila ortholog: Cyp307a2), CYP2C9, CYP2C19, CYP2D6, CYP2E1 (drosophila ortholog: Cyp18a1) and CYP3A4 (drosophila ortholog: Cyp9f2) in concentration ranges corresponding to their endogenous abundance in the liver^20^. When we incubated PCB28 with the rhCYP cocktail, 3-OH-CB28 and 3´-OH-CB28 were readily detected and confirmed that the metabolic activation of PCB 28 in vivo can be reproduced in vitro (Fig. 3A). Next, we divided the rhCYP cocktail into fractions and performed single rhCYP enzyme incubations to see which specific enzymes were responsible for the metabolic activation of PCB 28 in the rhCYP cocktail (Fig. 3B,C). All of the enzymatic activity on PCB 28 was found in the fraction containing CYP1A2, CYP2E1 and CYP3A4 (Fig. 3B). Particularly CYP1A2 as a single enzyme generated high levels of the OH-CB28 metabolites (Fig. 3C)^26^. This confirmed CYP1A2 as a central CYP in the formation of reactive OH-metabolites from PCB 28. Since CYP1A2 is a human orthologue of *Drosophila* Cyp307a2, these results indicate a conservation at the level of activity against PCB28 between *Drosophila melanogaster* and the human system.

### PCB 28 induces caspase activation in fly neurons

**Figure 4:**
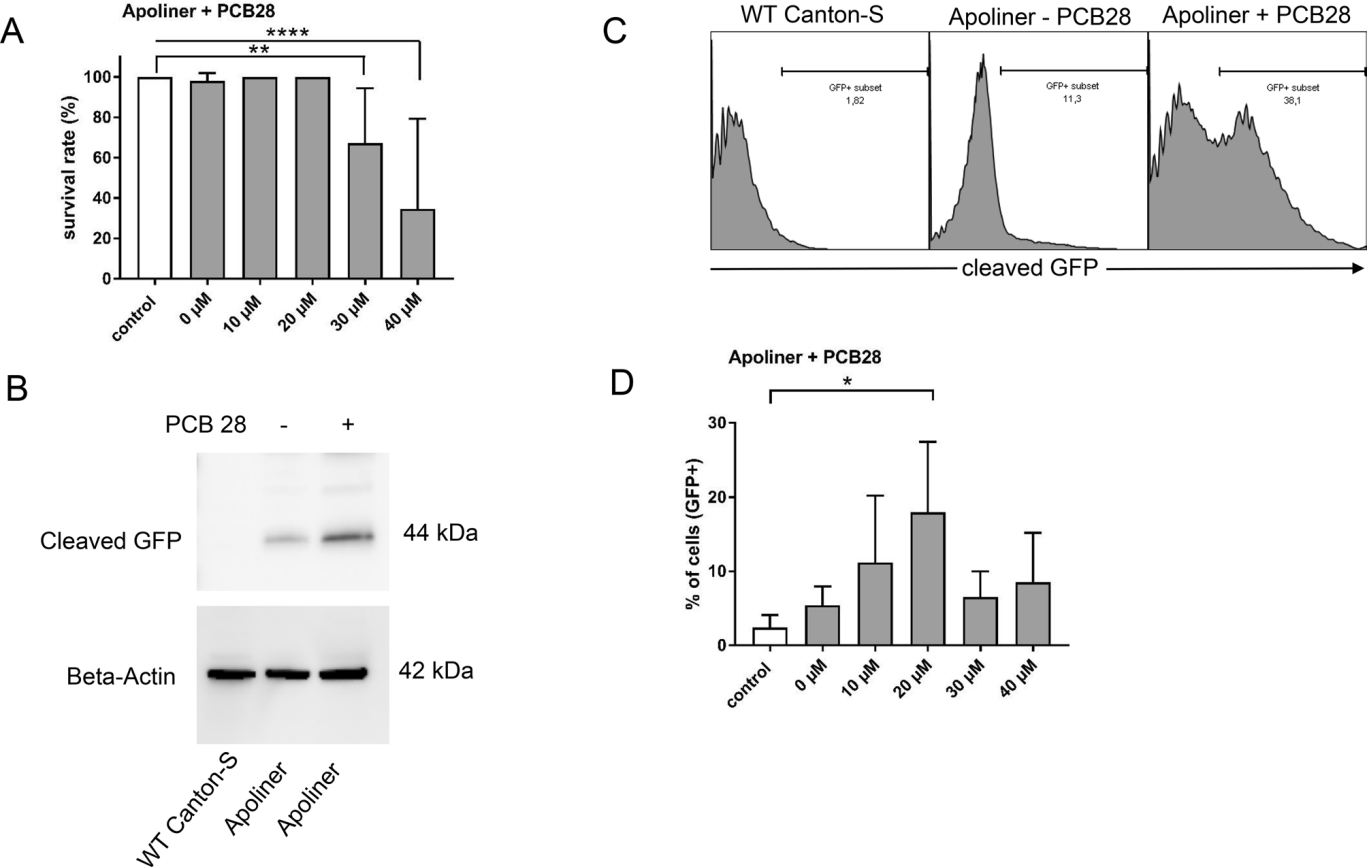
PCB 28 induces neuronal caspase activation prior to cell death and dying in *Drosophila*. (**A**) Wild type Canton-S (control, 0 µM PCB 28) and flies with a neuronal specific expression of the Apoliner biosensor were fed PCB 28 with the subsequent analysis of dead flies 24 h later. Mean ± SD of four different biological replicates each with a total of 12 flies are shown. (**B**) Western blot analysis of an apoptosis indicating GFP-containing fragment at 44 kDa after 4 h of feeding Apoliner expressing flies with PCB 28. Representative result from one experiment out of three is shown. (**C**) Flow cytometry analysis of the same flies which had been used for Western-blotting described in (**B**). (**D**) Flow cytometric quantification of the GFP signal in controls and carriers of the neuronally expressed biosensor. Mean values and standard deviation of 4 biological replicas, each with a total of 12 flies per replicate are shown. Differences were calculated by ANOVA and adjusted by posthoc Tukey-test for multiple measures. Statistical differences are as indicated (*p ≤ 0.05; **p ≤ 0,001; ****p ≤ 0.0001).

Administration of highly doses of PCB 28 induced early mortality in flies. Prior to death, flies displayed phenotypes like abnormal wing posture and reduced locomotion, typically observed in *Drosophila* models of neurodegenerative diseases^27^. In addition, neurotoxic effects of PCBs have been described in the literature^28–30^. We therefore focused on the analysis of PCB 28-induced cell death in the neuronal system, which can be executed by caspases. For visualization, we used a caspase-sensitive Apoliner biosensor, which indicates caspase activation. Apoliner consists of a membrane bound fused Red Fluorescent Protein (RFP) fused to an Enhanced Green Fluorescent Protein (eGFP) with a nuclear localization signal^31^. RFP and eGFP are separated by a linker, which contains a caspase cleavage side. Accordingly, this biosensor enables quantification of caspase activity as a precursor of apoptosis by detecting separation of GFP and RFP. By subjecting F1 generations to PCB 28, survival rate was reduced starting at a PCB 28 agar concentration of 30 µM (Fig. 4A). At an agar concentration of 40 µM only 34.6% of the flies survived on average (p = 0.026). In short-term incubations (4 h), PCB 28 induced an increase at the amount of cleaved GFP-containing fragments compared to PCB-free preparations and non-Apoliner strains (Fig. 4B and supplementary Fig. 1). The increase of cleaved GFP-containing fragments was paralleled to the appearance of a strong GFP fluorescent signal that could be analyzed by flow cytometry (Fig. 4C). Remarkably, only a weak GFP signal could be detected in flies not fed with PCB 28. We interpret that quenching by fluorescence resonance energy transfer (FRET) in non-apoptotic cells occurs through the interaction of the membrane-bound GFP and RFP and that the cleavage of the GFP-containing fragment during caspase activation also leads to GFP emitting its characteristic fluorescence. Flow cytometry thus provided an easy and fast way to analyze PCB 28-induced apoptosis in Apoliner strains. We have therefore performed a flow cytometry screening in flies with pan neural and retinal expression of Apoliner kept for 4 h on agar with increasing PCB 28 concentrations (Fig. 4D): flies showed a concentration-dependent increase of the GFP signal up to a PCB 28 concentration of 20 µM (average 15.6%), with a decrease of fluorescence at concentrations of 30 and 40 µM. This result correlated well with the results of the lethality screen (Fig. 4A), since an increase in lethality can be observed from a concentration of 30 µM PCB 28—probably as a consequence of neuronal apoptosis. In summary, we provide evidence that PCB 28 induces lethality in *Drosophila.* This PCB 28 dependent lethality could be a consequence of neuronal decline by apoptosis. Accordingly, PCB 28 exposure may resemble mechanisms of neurodegenerative diseases.

### Neurofilament light chain concentrations in PCB exposed individuals

**Figure 5:**
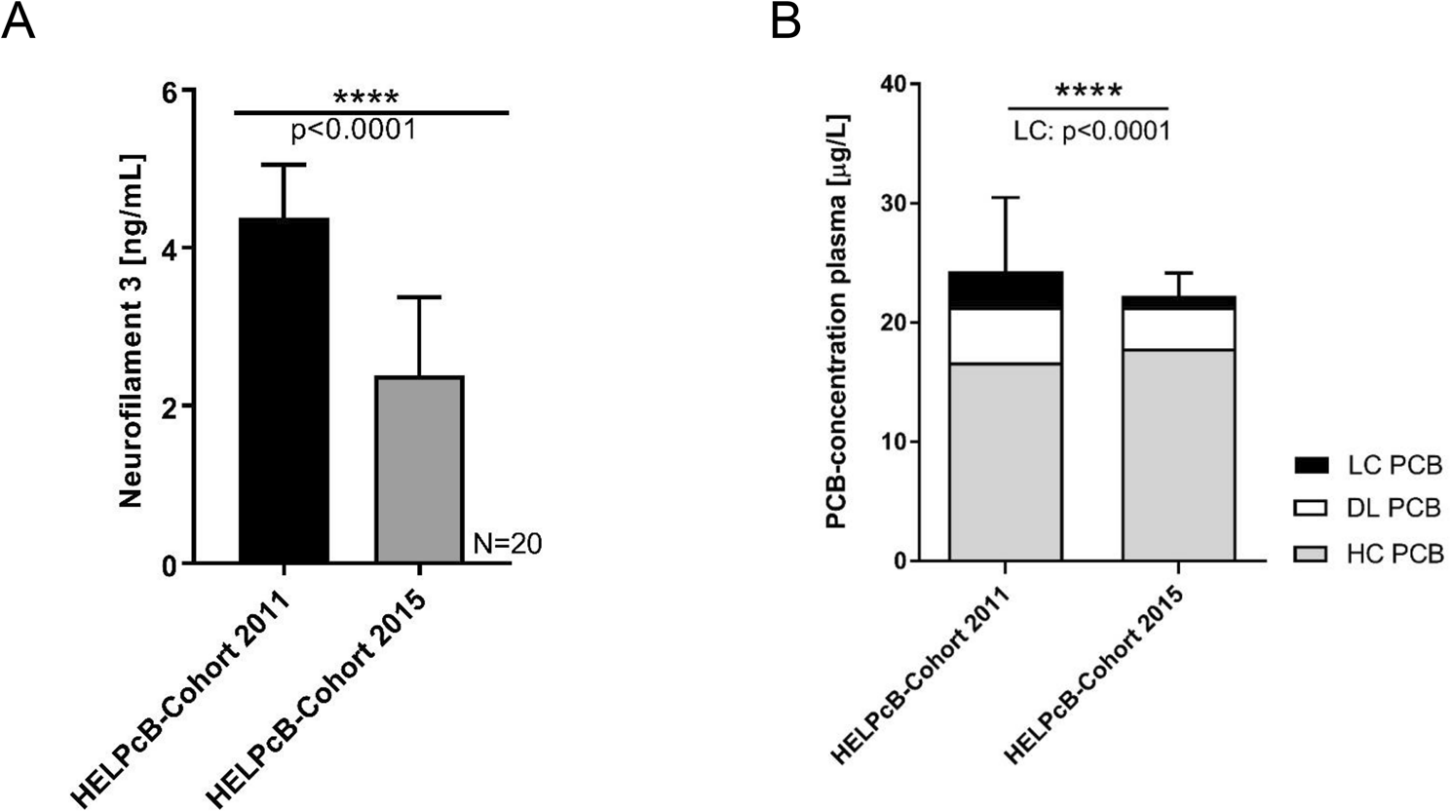
Neurofilament light chain levels in PCB exposed individuals. (**A**) Quantification of the neurofilament light chain in longitudinally collected plasma samples from the HELPcB-Cohort (N = 20 per time point; each with two technical replicates). (**B**) Mean plasma levels for Σ higher chlorinated PCBs (grey), Σ dioxin-like PCBs (white) and lower chlorinated PCBs (black) from longitudinally collected samples (HELPcB-Cohort). Differences were calculated by ANOVA and adjusted by posthoc Tukex-test for multiple measures. Statistical differences are as indicated ****p ≤ 0.0001).

In our recent work within the medical monitoring programme HELPcB (Health Effects in High-Level Exposure to PCBs)^32^, we were able to establish a correlation between exposure to LC PCBs and the concentrations of the metabolite homovanilic acid (HVA, metabolite of dopamine) in the urine of individuals^33^. A high exposure with lower chlorinated PCBs lead to an increase of urinary concentrations of HVA suggesting an impeding effect of LC PCBs on the dopamine neurotransmitter system. The dysfunction of the dopaminergic system is associated with various neurological disorders that are often accompanied by deleterious changes in cellular homeostasis and can lead to different types of regulated cell death in the cerebral tissue. Since our experiments in *Drosophila* suggested a caspase-mediated apoptosis in the nervous system induced by PCBs, we decided to search for signs of neurodegeneration in the HELPcB cohort. Therefore we determined the concentration of the neurofilament light chain (Nfl) in the plasma of PCB exposed individuals. Nfl is a biomarker that is released when axons are damaged or lost and is determined in a variety of neurodegenerative diseases^34,35^. When incubated with longitudinally collected blood plasma samples, the concentrations of Nfl dropped from its initial level (4.38 ± 0.67 ng/mL) in 2011 to a lower level (2.38 ± 0.98 ng/mL) in 2015 (Fig. 5A). Corresponding mean plasma levels of longitudinally collected samples of this cohort showed no significant difference in the level of higher chlorinated PCBs (HC PCBs) at two different time points (mean concentration HC PCB 2011 = 16.67 μg/L vs mean concentration HC PCB 2015 = 17.81 μg/L); whereas levels of dioxin-like PCBs (DL PCBs, mean concentration DL PCBs 2011 = 4.65 μg/L vs mean concentration DL PCBs 2015 = 3.5 μg/L) and lower chlorinated PCBs (LC PCBs, mean concentration LC PCBs 2011 = 2.98 μg/L vs mean concentration LC PCBs 2015 = 0.92 μg/L; p ≤ 0.0001) decreased over time (Fig. 5B). We, therefore, conclude that high LC PCB plasma levels increase axonal degeneration, thereby releasing higher concentrations of Nfl. Axonal degenerations is relieved over time with lower concentrations of Nfl in plasma samples from the HELPcB-Cohort, simultaneously to the decrease in levels LC PCBs. PCBs thus appear to cause irreversible cell damage in the nervous system both in humans and in *Drosophila*. This finding underlines that *Drosophila* has established itself as an interesting model to study PCB-induced neurotoxicity.

## Discussion

This study demonstrates that the degradation of PCB 28, PCB 101 and PCB 52 in *Drosophila melanogaster* produces the same pattern of OH-metabolites as the degradation of these lower chlorinated PCBs in humans. Metabolization takes place by cytochrome P450 (CYP) enzymes, which are very well conserved between invertebrates and vertebrates. Selected Cyps in *Drosophila* were specifically knocked down by means of RNA interference (RNAi) in order to subsequently investigate the effects on the conversion of PCB 28 in a metabolism screening. Lysates were produced from vital flies by mechanical and enzymatic digestion to investigate the metabolization rate of PCB 28 into its OH-metabolites using GC/MS and HPLC/MS/MS. Cyp307a2, 18a1 and 312a1 could be identified, whose knockdown caused the toxic metabolites 3-OHCB 28 and 3´-OHCB 28 to disappear and led to survival at high agar concentrations of PCB 28. In vitro, PCB 28 could be converted into all OH-metabolites by the combination of six recombinant human cytochromes (CYP1A2, CYP2C9, CYP2C19, CYP2D6, CYP2E1, CYP3A4), which are strongly expressed in the liver and thus reflect its metabolic properties. This result confirmed CYP1A2 as a central CYP in the formation of reactive OH-metabolites from PCB 28, since CYP1A2 contained in the liver-like rhCYP cocktail is a human orthologue of Cyp307a2 in *Drosophila*^26^. The feeding of *Drosophila* with PCB 28 led to neurotoxic signs and concentration-dependent lethality. By Western-blotting and flow cytometry this could be attributed to a PCB 28-dependent induction of apoptosis in differentiated neurons.

In recent years, various approaches have been developed in *Drosophila* to characterize the role of CYPs in the metabolism of endogenous and exogenous compounds^36,37^. In addition, *Drosophila* has been established as a model for human neurodegenerative diseases^38–41^. These models are used to investigate genetic causes for neurodegenerative processes and the genetic and environmental risk factors associated with Parkinson's disease^42^. In this context, it was found that the basic cell biological signal transduction pathways between invertebrates and vertebrates are surprisingly well preserved. This is also evident when comparing the fully sequenced genomes of both organisms. In addition, established genetics, small body size, short generation time and lifespan characterize *Drosophila* as a model organism, especially for large genetic screens. Our study shows that the similarities between invertebrates and vertebrates could be used to study xenobiotic PCB metabolism as well as PCB-induced neurotoxicity and generate data relevant to humans.

Insect genomes possess about 100 different Cyp isozymes, many of which have no essential endogenous function, which is considered an evolutionary adaptation to the large number of harmful compounds in the insects environment^37,43^. The *Drosophila* genome codes for apparently functional 90 Cyp genes. More than half of the genes belong to only two families, Cyp4 and Cyp6. The Cyp6 family is insect specific whereas the Cyp4 family includes sequences from vertebrates. Although *Drosophila* does not possess the human CYP enzyme superfamilies 1–3, it nevertheless possesses corresponding genetically related orthologous enzymes from this family. The Cyp307a2 identified in our screen has an essential endogenous function in *Drosophila* and its putative substrate recognition sites are conserved across vertebrate orthologous genes^37,44^.

The CYP system is at the onset of xenobiotic metabolism and plays a key role in the bioaccumulation and toxicity of PCBs*.* Feeding experiments on rats with commercially available PCB mixtures such as Aroclor 1254, Fire-Master Bp-6 or individual congeners showed the induction of different CYPs depending on the position of the chlorine atoms on the phenyl ring^45^: Non-orthochlorinated coplanar PCBs such as PCB 126, PCB 169 and PCB 77 induce the expression of CYP1A1 and CYP1A2. Orthochlorinated PCBs such as PCB 128, PCB 153, PCB 155 and PCB 180 induce the expression of CYP2B1. Other structured PCBs such as PCB 118, PCB 138 and PCB 170 induce the expression of both CYP1A1 and CYP2B^45^. Some of these CYPs could be partially confirmed as actual catalysts of hydroxylation of these PCBs in vitro^46–49^. In addition, the hydroxylation of some tri- and tetrachlorobiphenyls (PCB 20, 22, 52 and 74) by CYP2E1^50^ and the formation of 4-OHCB 101 from PCB 101 by CYP2A6 was detected in vitro^51^. The human CYP1A2, identified in this study is capable of oxidizing estradiol, resulting in the production of 2-hydroxy and 4-hydroxy metabolites^52^. Xenobiotic substrates of CYP1A2 include caffeine^53^, aflatoxin B1^54^ and paracetamol^55^. It has been suggested that the increased function of CYP1A2 may be associated with an increased risk of breast cancer^56^, which could ultimately also be important for the metabolism of PCBs: In individual cases, existing exposure of PCBs in conjunction with genetic polymorphisms in CYP1A2 could lead to an increase in biological efficacy of PCBs (toxicity/mutagenicity), which could also result in an increased risk of disease. Epidemiological results indicate that the cumulative exposure dose is not the only relevant risk measure for the effect of PCBs^57^. Therefore, in further steps, CYP1A2 variants might be identified, that accelerate the metabolism of PCB 28 in humans.

We show here for PCB 28 the concentration-dependent induction of cell death in differentiated neurons in vivo*.* PCBs have also been shown to induce caspase-dependent cell death in embryonic rat hippocampal cells via the ryanodine receptor in vitro^58^. Other proposed mechanisms of PCB neurotoxicity include altered neurotransmitters and calcium homeostasis, oxidative stress and effects on the thyroid hormone system^59^. The effects described for PCBs on humans, such as axonal polyneuropathies and effects on memory function and fine motor skills in adults and children, could thus also be due to the loss of neuronal cells^15–17^.

Our results find a correspondence in the action of rotenone and paraquat, both environmental toxins, which have been used by humans for decades: rotenone and paraquat are able to cause cell loss in the dopaminergic system and are used in models of sporadic Parkinson's disease in *Drosophila*^27,39,60^. More publications show experimental evidence for paraquat-induced apoptosis by JNK-MAPK-induced caspases^61,62^ and include an increase in oxidative stress triggered by dysfunctional mitochondria. However, we have not found any other insect reports that provide a global picture of CYP-related metabolism and support the neurotoxic effects of industrial solvents. In fact, only one study considers the CYP-dependent metabolism of a xenobiotic compound that leads to its degradation in *Drosophila melanogaster*: After feeding flies with caffeine, Coelho and colleagues identified theobromine, paraxanthin and theophylline, three metabolites derived from Drosophila caffeine that also occur in mammals^36^. Provided that the similarity in metabolism between humans and Drosophila can be replicated for other classes of chemicals, this and our study could pave the way to a better understanding of the degradation mechanism and toxicity of xenobiotic compounds in the human body.

## Supplementary Information

Supplementary Information
