# Supplementary material for: Metabolic activation and toxicological evaluation of polychlorinated biphenyls in Drosophila melanogaster
Source: Sci Rep. 2020 Dec 9;10:21587. doi: 10.1038/s41598-020-78405-z (PMC7726022; doi:10.1038/s41598-020-78405-z)
Supplement: Supplementary file 1 — Supplementary Information [file 41598_2020_78405_MOESM1_ESM.docx]

**Supplementary information to:**

**Metabolic activation and toxicological evaluation of polychlorinated biphenyls in *Drosophila melanogaster***

Idda T^1*^, Bonas C^1*^, Hoffmann J^1^, Bertram J^1^, Quinete N^1,2^, Schettgen T^1^, Fietkau K^3^, Esser A^1^, Stope MB^4^, Leijs MM^3^, Baron JM^3^, Kraus T^1^, Voigt A^5,6^, Ziegler P^1^

1. Institute for Occupational, Social and Environmental Medicine, RWTH Aachen University, Aachen, Germany.

2. Department of Chemistry and Biochemistry, Florida International University Florida, Florida, USA.

3. Department of Dermatology and Allergology, RWTH Aachen University, 52074 Aachen, Germany.

4. Department of Gynecology and Gynecological Oncology, University Hospital Bonn,

Germany

5. Department of Neurology, University Medical Center, RWTH Aachen University, Aachen, 52074, Germany

6. JARA-BRAIN Institute Molecular Neuroscience and Neuroimaging, Forschungszentrum Jülich GmbH and RWTH Aachen University, 52074 Aachen, Germany

* These authors contributed equally

Correspondence to: pziegler@ukaachen.de

**Supplementary Fig. 1:** Full-length Western Blot GFP- containing fragments . Exposure time: 30 seconds.

Experiment 1


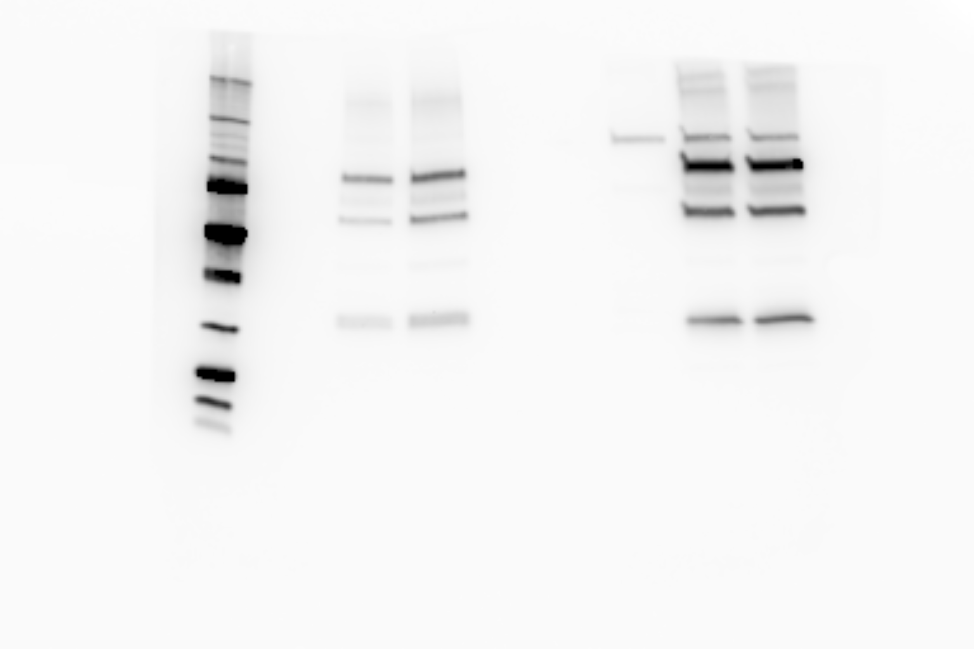
PCB28: - +

1: Marker

2: WT Canton-S

3: Apoliner

4: Apoliner

PCB28: - +

44 kDa

1 2 3 4

Experiment 2

PCB28: - +


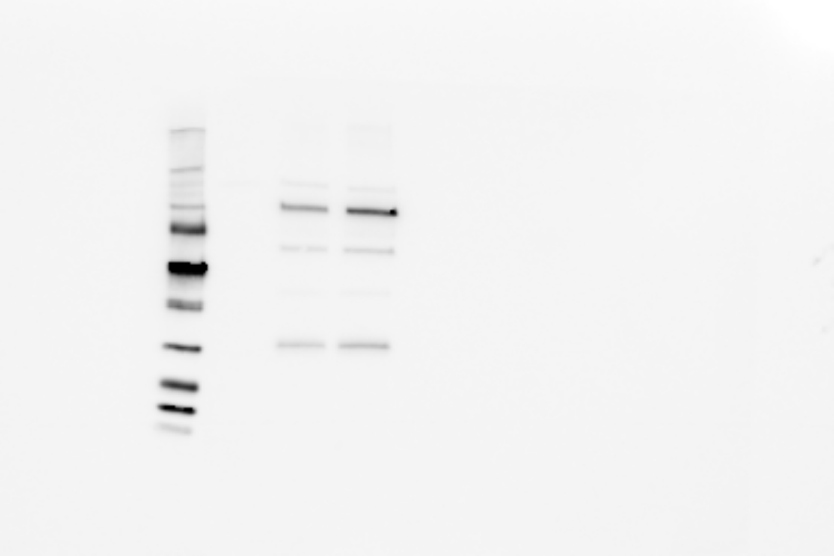


1: Marker

2: WT Canton-S

3: Apoliner

4: Apoliner

44 kDa

1 2 3 4

Experiment 3

PCB28: - +


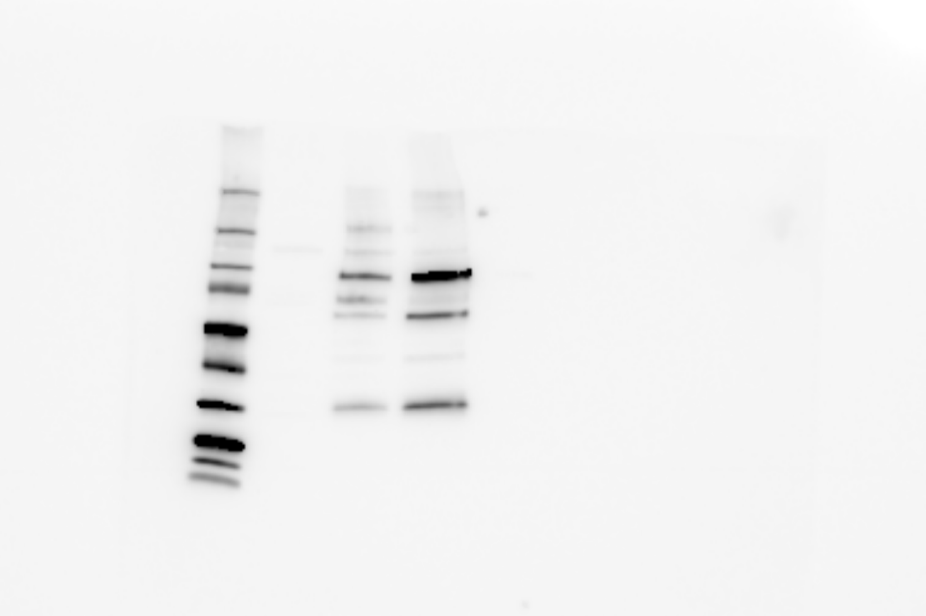


1: Marker

2: WT Canton-S

3: Apoliner

4: Apoliner

44 kDa

1 2 3 4

**Supplementary Fig. 2** Full-length Western Blot β-Actin (stripped after GFP detection). Exposure time: 150 seconds.

Experiment 1

PCB28: - +


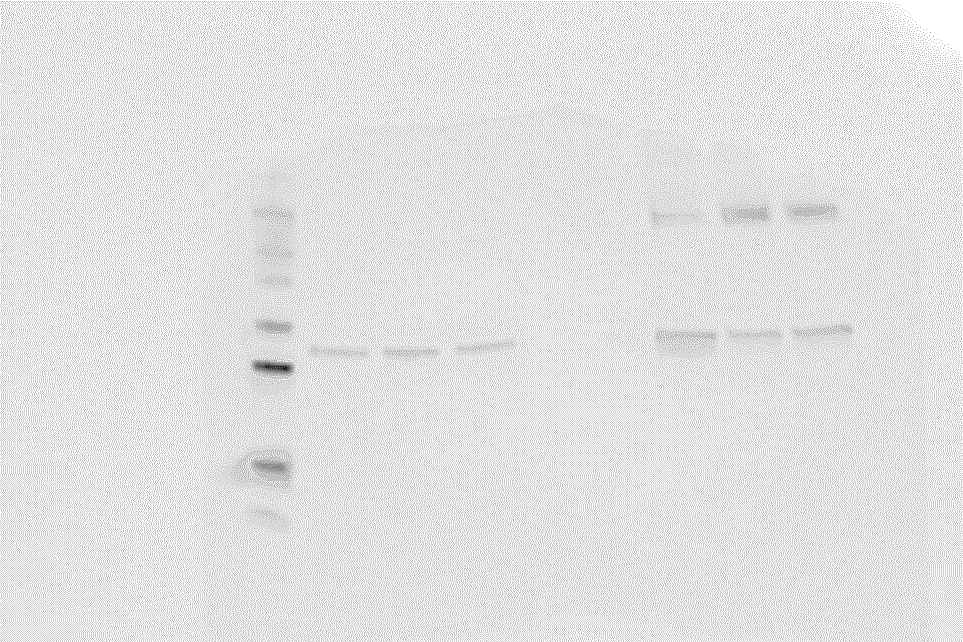


1: Marker

2: WT Canton-S

3: Apoliner

4: Apoliner

1 2 3 4

42 kDa

Experiment 2

PCB28: - +


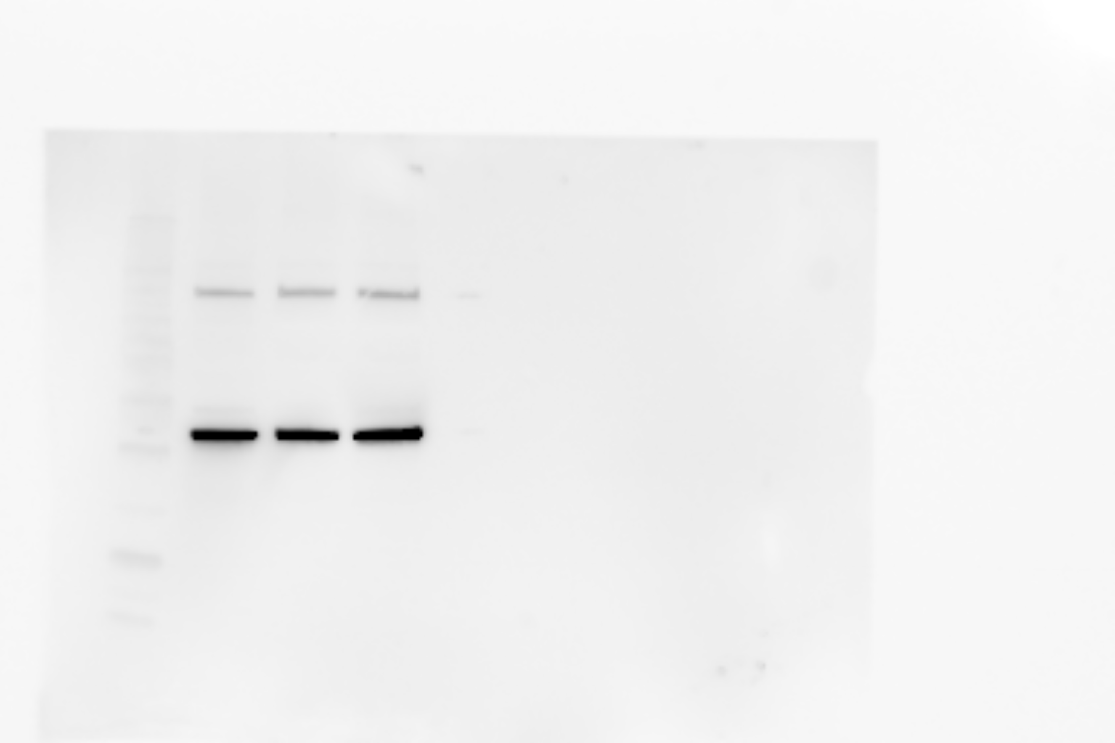


1: Marker

2: WT Canton-S

3: Apoliner

4: Apoliner

42 kDa

Experiment 3

PCB28: - +


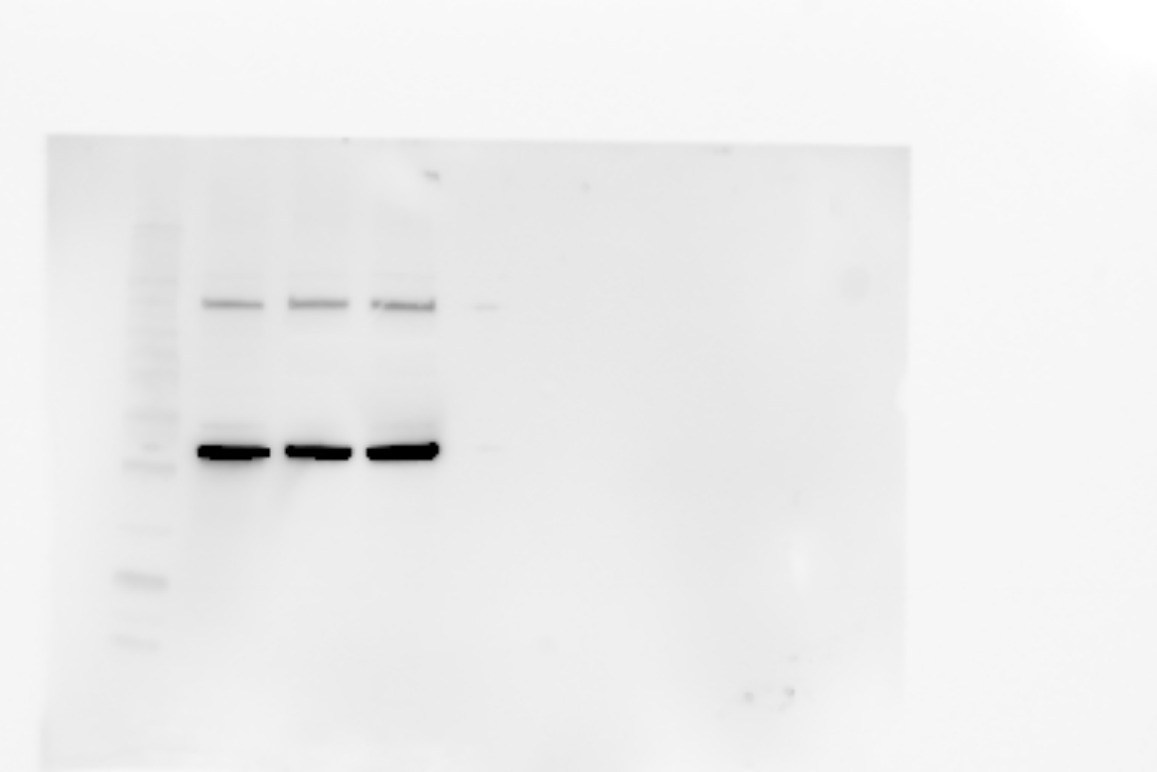


1: Marker

2: WT Canton-S

3: Apoliner

4: Apoliner

42 kDa

**Supplementary Fig. 3** Results of the statistical analysis

**Corresponding Figure 1C** (survival) 48h. Differences were calculated by repeated measurement ANOVA and posthoc Tukey-test

| **Differences of medium Least Squares Means Adjustment for Multiple Comparisons: Tukey** | | | | | | | |
| --- | --- | --- | --- | --- | --- | --- | --- |
| **medium** | **medium** | **Estimation** | **Standard Error** | **DF** | **t** | **Pr > \|t\|** | **Adj P** |
| **PCB101** | **PCB28** | 63.8867 | 7.3486 | 8 | 8.69 | <.0001 | 0.0001 |
| **PCB101** | **PCB52** | 2.7767 | 7.3486 | 8 | 0.38 | 0.7154 | 0.9803 |
| **PCB101** | **control** | 3.55E-15 | 7.3486 | 8 | 0.00 | 1.0000 | 1.0000 |
| **PCB28** | **PCB52** | -61.1100 | 7.3486 | 8 | -8.32 | <.0001 | 0.0002 |
| **PCB28** | **control** | -63.8867 | 7.3486 | 8 | -8.69 | <.0001 | 0.0001 |
| **PCB52** | **control** | -2.7767 | 7.3486 | 8 | -0.38 | 0.7154 | 0.9803 |

**Corresponding Figure 1C** (survival) 72h. Differences were calculated by repeated measurement ANOVA and posthoc Tukey-test

| **Differences of medium Least Squares Means Adjustment for Multiple Comparisons: Tukey** | | | | | | | |
| --- | --- | --- | --- | --- | --- | --- | --- |
| **medium** | **medium** | **Estimation** | **Standard Error** | **DF** | **t** | **Pr > \|t\|** | **Adj P** |
| **PCB101** | **PCB28** | 77.7767 | 8.5618 | 8 | 9.08 | <.0001 | <.0001 |
| **PCB101** | **PCB52** | 2.7767 | 8.5618 | 8 | 0.32 | 0.7540 | 0.9873 |
| **PCB101** | **control** | -5.5567 | 8.5618 | 8 | -0.65 | 0.5345 | 0.9130 |
| **PCB28** | **PCB52** | -75.0000 | 8.5618 | 8 | -8.76 | <.0001 | 0.0001 |
| **PCB28** | **control** | -83.3333 | 8.5618 | 8 | -9.73 | <.0001 | <.0001 |
| **PCB52** | **control** | -8.3333 | 8.5618 | 8 | -0.97 | 0.3589 | 0.7679 |

Corresponding Figure 2A (3-OHCB28). Differences were calculated by repeated measurement ANOVA and posthoc Tukey-test

| **Differences of FLY Least Squares Means Adjustment for Multiple Comparisons: Tukey** | | | | | | | |
| --- | --- | --- | --- | --- | --- | --- | --- |
| **FLY** | **FLY** | **Estimation** | **Standard Error** | **DF** | **t** | **Pr > \|t\|** | **Adj P** |
| **CYP18a1** | **CYP307A2** | 0.1033 | 0.08019 | 11 | 1.29 | 0.2240 | 0.5880 |
| **CYP18a1** | **CYP312a1** | -0.00333 | 0.08019 | 11 | -0.04 | 0.9676 | 1.0000 |
| **CYP18a1** | **WT control** | -0.00333 | 0.08019 | 11 | -0.04 | 0.9676 | 1.0000 |
| **CYP307A2** | **CYP312a1** | -0.1067 | 0.09259 | 11 | -1.15 | 0.2738 | 0.6671 |
| **CYP307A2** | **WT control** | -0.1067 | 0.09259 | 11 | -1.15 | 0.2738 | 0.6671 |
| **CYP312a1** | **WT control** | -278E-19 | 0.09259 | 11 | -0.00 | 1.0000 | 1.0000 |

Corresponding Figure 2B (3´-OHCB28). Differences were calculated by repeated measurement ANOVA and posthoc Tukey-test

| **Differences of FLY Least Squares Means Adjustment for Multiple Comparisons: Tukey-Kramer** | | | | | | | |
| --- | --- | --- | --- | --- | --- | --- | --- |
| **FLY** | **FLY** | **Estimation** | **Standard Error** | **DF** | **t** | **Pr > \|t\|** | **Adj P** |
| **CYP18a1** | **CYP307A2** | -0.1633 | 0.4972 | 11 | -0.33 | 0.7487 | 0.9871 |
| **CYP18a1** | **CYP312a1** | 0.2767 | 0.4972 | 11 | 0.56 | 0.5890 | 0.9428 |
| **CYP18a1** | **WT control** | -1.3633 | 0.4972 | 11 | -2.74 | 0.0192 | 0.0777 |
| **CYP307A2** | **CYP312a1** | 0.4400 | 0.5741 | 11 | 0.77 | 0.4596 | 0.8678 |
| **CYP307A2** | **WT control** | -1.2000 | 0.5741 | 11 | -2.09 | 0.0606 | 0.2154 |
| **CYP312a1** | **WT control** | -1.6400 | 0.5741 | 11 | -2.86 | 0.0156 | 0.0644 |

Corresponding Figure 2C (survival). Differences were calculated by repeated measurement ANOVA and posthoc Tukey-test

| **Differences of FLY Least Squares Means Adjustment for Multiple Comparisons: Tukey-Kramer** | | | | | | | |
| --- | --- | --- | --- | --- | --- | --- | --- |
| **FLY** | **FLY** | **Estimation** | **Standard Error** | **DF** | **t** | **Pr > \|t\|** | **Adj P** |
| **CYP18a1** | **CYP307A2** | 0.02899 | 0.09371 | 7 | 0.31 | 0.7660 | 0.9888 |
| **CYP18a1** | **CYP312a1** | 0.02899 | 0.09371 | 7 | 0.31 | 0.7660 | 0.9888 |
| **CYP18a1** | **WT control** | 1.5889 | 0.1048 | 7 | 15.17 | <.0001 | <.0001 |
| **CYP307A2** | **CYP312a1** | 0 | 0.09371 | 7 | 0.00 | 1.0000 | 1.0000 |
| **CYP307A2** | **WT control** | 1.5599 | 0.1048 | 7 | 14.89 | <.0001 | <.0001 |
| **CYP312a1** | **WT control** | 1.5599 | 0.1048 | 7 | 14.89 | <.0001 | <.0001 |

Corresponding Figure 3B. Differences were calculated by repeated measurement ANOVA and posthoc Tukey-test

## 3-OH-PCB 28

| **Differences of CYP_group Least Squares Means Adjustment for Multiple Comparisons: Tukey-Kramer** | | | | | | | |
| --- | --- | --- | --- | --- | --- | --- | --- |
| **CYP-group** | **CYP-group** | **Estimation** | **Standard Error** | **DF** | **t** | **Pr > \|t\|** | **Adj P** |
| **GREY** | **WHITE** | 0.08000 | 0.08000 | 4 | 1.00 | 0.3739 | 0.3739 |

## 4-OH/4´-OH_CB28

| **Differences of CYP_group Least Squares Means Adjustment for Multiple Comparisons: Tukey-Kramer** | | | | | | | |
| --- | --- | --- | --- | --- | --- | --- | --- |
| **CYP-group** | **CYP-group** | **Estimation** | **Standard Error** | **DF** | **t** | **Pr > \|t\|** | **Adj P** |
| **GREY** | **WHITE** | 0.2633 | 0.09098 | 4 | 2.89 | 0.0444 | 0.0444 |

## 3´-OH_CB28

| **Differences of CYP_group Least Squares Means Adjustment for Multiple Comparisons: Tukey-Kramer** | | | | | | | |
| --- | --- | --- | --- | --- | --- | --- | --- |
| **CYP-group** | **CYP-group** | **Estimation** | **Standard Error** | **DF** | **t** | **Pr > \|t\|** | **Adj P** |
| **GREY** | **WHITE** | 2.2767 | 0.08333 | 4 | 27.32 | <.0001 | <.0001 |

Corresponding Figure 3C. Differences were calculated by repeated measurement ANOVA and posthoc Tukey-test

## 3-OH_CB28

| **Differences of CYP Least Squares Means Adjustment for Multiple Comparisons: Tukey** | | | | | | | |
| --- | --- | --- | --- | --- | --- | --- | --- |
| **CYP** | **CYP** | **Estimation** | **Standard Error** | **DF** | **t** | **Pr > \|t\|** | **Adj P** |
| **CYP1A2** | **CYP2E1** | 0.1300 | 0.1020 | 6 | 1.27 | 0.2495 | 0.4577 |
| **CYP1A2** | **CYP3A4** | 0.1333 | 0.1020 | 6 | 1.31 | 0.2389 | 0.4418 |
| **CYP2E1** | **CYP3A4** | 0.003333 | 0.1020 | 6 | 0.03 | 0.9750 | 0.9994 |

## 4-OH/4´-OH_CB28

| **Differences of CYP Least Squares Means Adjustment for Multiple Comparisons: Tukey** | | | | | | | |
| --- | --- | --- | --- | --- | --- | --- | --- |
| **CYP** | **CYP** | **Estimation** | **Standard Error** | **DF** | **t** | **Pr > \|t\|** | **Adj P** |
| **CYP1A2** | **CYP2E1** | 0.3133 | 0.02373 | 6 | 13.21 | <.0001 | <.0001 |
| **CYP1A2** | **CYP3A4** | 0.2733 | 0.02373 | 6 | 11.52 | <.0001 | <.0001 |
| **CYP2E1** | **CYP3A4** | -0.04000 | 0.02373 | 6 | -1.69 | 0.1428 | 0.2849 |

## 3´-OH_CB28

| **Differences of CYP Least Squares Means Adjustment for Multiple Comparisons: Tukey** | | | | | | | |
| --- | --- | --- | --- | --- | --- | --- | --- |
| **CYP** | **CYP** | **Estimation** | **Standard Error** | **DF** | **t** | **Pr > \|t\|** | **Adj P** |
| **CYP1A2** | **CYP2E1** | 3.4733 | 0.1695 | 6 | 20.49 | <.0001 | <.0001 |
| **CYP1A2** | **CYP3A4** | 3.4400 | 0.1695 | 6 | 20.29 | <.0001 | <.0001 |
| **CYP2E1** | **CYP3A4** | -0.03333 | 0.1695 | 6 | -0.20 | 0.8506 | 0.9790 |

Corresponding Figure 4A. Differences were calculated by repeated measurement ANOVA and posthoc Tukey-test

| **Differences of treatment Least Squares Means Adjustment for Multiple Comparisons: Tukey** | | | | | | | |
| --- | --- | --- | --- | --- | --- | --- | --- |
| **treatment** | **treatment** | **Estimation** | **Standard Error** | **DF** | **t** | **Pr > \|t\|** | **Adj P** |
| **0 µM** | **10 µM** | -0.01941 | 0.07106 | 18 | -0.27 | 0.7878 | 0.9998 |
| **0 µM** | **20 µM** | -0.01941 | 0.07106 | 18 | -0.27 | 0.7878 | 0.9998 |
| **0 µM** | **30 µM** | 0.3777 | 0.07917 | 18 | 4.77 | 0.0002 | 0.0018 |
| **0 µM** | **40 µM** | 1.0415 | 0.09885 | 18 | 10.54 | <.0001 | ***<.0001*** |
| **0 µM** | **control** | -0.01941 | 0.07106 | 18 | -0.27 | 0.7878 | 0.9998 |
| **10 µM** | **20 µM** | 2.67E-17 | 0.07071 | 18 | 0.00 | 1.0000 | 1.0000 |
| **10 µM** | **30 µM** | 0.3971 | 0.07886 | 18 | 5.04 | <.0001 | ***0.0010*** |
| **10 µM** | **40 µM** | 1.0609 | 0.09860 | 18 | 10.76 | <.0001 | ***<.0001*** |
| **10 µM** | **control** | 2.7E-16 | 0.07071 | 18 | 0.00 | 1.0000 | 1.0000 |
| **20 µM** | **30 µM** | 0.3971 | 0.07886 | 18 | 5.04 | <.0001 | ***0.0010*** |
| **20 µM** | **40 µM** | 1.0609 | 0.09860 | 18 | 10.76 | <.0001 | ***<.0001*** |
| **20 µM** | **control** | 2.44E-16 | 0.07071 | 18 | 0.00 | 1.0000 | 1.0000 |
| **30 µM** | **40 µM** | 0.6638 | 0.1046 | 18 | 6.35 | <.0001 | ***<.0001*** |
| **30 µM** | **control** | -0.3971 | 0.07886 | 18 | -5.04 | <.0001 | ***0.0010*** |
| **40 µM** | **control** | -1.0609 | 0.09860 | 18 | -10.76 | <.0001 | ***<.0001*** |
|  |  |  |  |  |  |  |  |

Corresponding Figure 4D. Differences were calculated by repeated measurement ANOVA and posthoc Tukey-test

| **Differences of treatment Least Squares Means Adjustment for Multiple Comparisons: Tukey-Kramer** | | | | | | | |
| --- | --- | --- | --- | --- | --- | --- | --- |
| **treatment** | **treatment** | **Schätzung** | **Standard Error** | **DF** | **t** | **Pr > \|t\|** | **Adj P** |
| **0 µM** | **10 µM** | -0.7259 | 0.3024 | 12 | -2.40 | 0.0335 | 0.2298 |
| **0 µM** | **20 µM** | -1.1999 | 0.2832 | 12 | -4.24 | 0.0012 | ***0.0114*** |
| **0 µM** | **30 µM** | -0.1897 | 0.3355 | 12 | -0.57 | 0.5823 | 0.9916 |
| **0 µM** | **40 µM** | -0.4569 | 0.3172 | 12 | -1.44 | 0.1754 | 0.7044 |
| **0 µM** | **control** | 0.8086 | 0.4471 | 12 | 1.81 | 0.0956 | 0.4955 |
| **10 µM** | **20 µM** | -0.4740 | 0.2199 | 12 | -2.16 | 0.0522 | 0.3238 |
| **10 µM** | **30 µM** | 0.5362 | 0.2842 | 12 | 1.89 | 0.0837 | 0.4534 |
| **10 µM** | **40 µM** | 0.2690 | 0.2624 | 12 | 1.03 | 0.3255 | 0.9006 |
| **10 µM** | **control** | 1.5345 | 0.4100 | 12 | 3.74 | 0.0028 | ***0.0262*** |
| **20 µM** | **30 µM** | 1.0102 | 0.2637 | 12 | 3.83 | 0.0024 | ***0.0226*** |
| **20 µM** | **40 µM** | 0.7430 | 0.2400 | 12 | 3.10 | 0.0093 | 0.0773 |
| **20 µM** | **control** | 2.0085 | 0.3961 | 12 | 5.07 | 0.0003 | ***0.0029*** |
| **30 µM** | **40 µM** | -0.2672 | 0.3000 | 12 | -0.89 | 0.3906 | 0.9416 |
| **30 µM** | **control** | 0.9983 | 0.4351 | 12 | 2.29 | 0.0406 | 0.2674 |
| **40 µM** | **control** | 1.2655 | 0.4211 | 12 | 3.01 | 0.0110 | 0.0898 |

Corresponding Figure 5A. Differences were calculated by repeated measurement ANOVA and posthoc Tukey-test

| **Differences of Year Least Squares Means Adjustment for Multiple Comparisons: Tukey** | | | | | | | |
| --- | --- | --- | --- | --- | --- | --- | --- |
| **Year** | **Year** | **Estimation** | **Standard Error** | **DF** | **t-** | **Pr > \|t\|** | **Adj P** |
| **HELPcB-Cohort 2011** | **HELPcB-Cohort 2015** | 2.0000 | 0.2665 | 38 | 7.50 | <.0001 | <.0001 |

Corresponding Figure 5B. Differences were calculated by repeated measurement ANOVA and posthoc Tukey-test

## PCB-sum

| **Differences of year Least Squares Means Adjustment for Multiple Comparisons: Tukey** | | | | | | | |
| --- | --- | --- | --- | --- | --- | --- | --- |
| **year** | **year** | **Estimation** | **Standard Error** | **DF** | **t-Wert** | **Pr > \|t\|** | **Adj P** |
| **HELPcB-Cohort 2011** | **HELPcB-Cohort 2015** | 0.1103 | 0.4352 | 38 | 0.25 | 0.8012 | 0.8012 |

## HC-PCB

| **Differences of year Least Squares Means Adjustment for Multiple Comparisons: Tukey** | | | | | | | |
| --- | --- | --- | --- | --- | --- | --- | --- |
| **year** | **year** | **Estimation** | **Standard Error** | **DF** | **t-Wert** | **Pr > \|t\|** | **Adj P** |
| **HELPcB-Cohort 2011** | **HELPcB-Cohort 2015** | -0.05564 | 0.4303 | 38 | -0.13 | 0.8978 | 0.8978 |

## DL-PCB

| **Differences of year Least Squares Means Adjustment for Multiple Comparisons: Tukey-Kramer** | | | | | | | |
| --- | --- | --- | --- | --- | --- | --- | --- |
| **year** | **year** | **Estimation** | **Standard Error** | **DF** | **t-Wert** | **Pr > \|t\|** | **Adj P** |
| **HELPcB-Cohort 2011** | **HELPcB-Cohort 2015** | 0.2844 | 0.1583 | 38 | 1.80 | 0.0804 | 0.0804 |

## LC-PCB

| **Differences of year Least Squares Means Adjustment for Multiple Comparisons: Tukey-Kramer** | | | | | | | |
| --- | --- | --- | --- | --- | --- | --- | --- |
| **year** | **year** | **Schätzung** | **Standard Error** | **DF** | **t-Wert** | **Pr > \|t\|** | **Adj P** |
| **HELPcB-Cohort 2011** | **HELPcB-Cohort 2015** | 1.1707 | 0.2664 | 38 | 4.39 | <.0001 | <.0001 |
